# Usefulness of Multiplex Real-Time PCR for Simultaneous Pathogen Detection and Resistance Profiling of Staphylococcal Bacteremia

**DOI:** 10.1155/2016/6913860

**Published:** 2016-06-15

**Authors:** Yousun Chung, Taek Soo Kim, Young Gi Min, Yun Ji Hong, Jeong Su Park, Sang Mee Hwang, Kyoung-Ho Song, Eu Suk Kim, Kyoung Un Park, Hong Bin Kim, Junghan Song, Eui-Chong Kim

**Affiliations:** ^1^Department of Laboratory Medicine, Seoul National University College of Medicine, 101 Daehak-ro, Jongno-gu, Seoul 03080, Republic of Korea; ^2^Department of Laboratory Medicine, Seoul National University Hospital, 101 Daehak-ro, Jongno-gu, Seoul 03080, Republic of Korea; ^3^Department of Laboratory Medicine, Seoul National University Bundang Hospital, 82 Gumi-ro, 173 Beon-gil, Bundang-gu, Seongnam-si, Gyeonggi-do 13620, Republic of Korea; ^4^Department of Internal Medicine, Seoul National University Bundang Hospital, 82 Gumi-ro, 173 Beon-gil, Bundang-gu, Seongnam-si, Gyeonggi-do 13620, Republic of Korea

## Abstract

Staphylococci are the leading cause of nosocomial blood stream infections. Fast and accurate identification of staphylococci and confirmation of their methicillin resistance are crucial for immediate treatment with effective antibiotics. A multiplex real-time PCR assay that targets* mecA*,* femA* specific for* S. aureus*,* femA* specific for* S. epidermidis*,* 16S rRNA* for universal bacteria, and* 16S rRNA* specific for staphylococci was developed and evaluated with 290 clinical blood culture samples containing Gram-positive cocci in clusters (GPCC). For the 262 blood cultures identified to the species level with the MicroScan WalkAway system (Siemens Healthcare Diagnostics, USA), the direct real-time PCR assay of positive blood cultures showed very good agreement for the categorization of staphylococci into methicillin-resistant* S. aureus* (MRSA), methicillin-susceptible* S. aureus* (MSSA), methicillin-resistant* S. epidermidis* (MRSE), methicillin-susceptible* S. epidermidis* (MSSE), methicillin-resistant non-*S. epidermidis* CoNS (MRCoNS), and methicillin-susceptible non-*S. epidermidis* CoNS (MSCoNS) (*κ* = 0.9313). The direct multiplex real-time PCR assay of positive blood cultures containing GPCC can provide essential information at the critical point of infection with a turnaround time of no more than 4 h. Further studies should evaluate the clinical outcome of using this rapid real-time PCR assay in glycopeptide antibiotic therapy in clinical settings.

## 1. Introduction

Staphylococci are the most commonly isolated organisms in clinical laboratories, accounting for almost 30% of all nosocomial infections and 50% of nosocomial bloodstream infections [1].* Staphylococcus aureus* is the leading cause of nosocomial infections [2], and methicillin-resistant* S. aureus* (MRSA) infections result in significant morbidity, mortality, and longer hospital stays if not treated early with effective antibiotics [3]. Coagulase-negative staphylococci (CoNS) are the most common isolates from blood culture, and more than 70% are resistant to oxacillin [4]. Although they are known to contaminate blood cultures as a result of their colonization on the skin and mucous membranes, they have recently become important pathogens causing nosocomial infections with the increasing use of invasive procedures and prosthetic devices [5, 6]. Fast and accurate identification of staphylococci and confirmation of their methicillin resistance are crucial for immediate treatment with effective antibiotics, which will result in decreased morbidity and mortality rates [7].

The conventional culture method for the identification and susceptibility testing of positive blood cultures has some disadvantages, including long turnaround time and potential false-negative results when samples are obtained after antimicrobial therapy. Real-time PCR is significantly faster than conventional PCR and other detection methods, and its excellent sensitivity and specificity, low contamination risk, ease of use, and high speed have made real-time PCR technology appealing to clinical microbiology laboratories [8].

The aim of this study was to develop and evaluate a multiplex real-time PCR assay for the rapid detection and identification of MRSA, methicillin-susceptible* S. aureus* (MSSA), methicillin-resistant* S. epidermidis* (MRSE), methicillin-susceptible* S. epidermidis* (MSSE), methicillin-resistant non*-S. epidermidis* CoNS (MRCoNS), and methicillin-susceptible non*-S. epidermidis* CoNS (MSCoNS) directly from positive blood cultures containing Gram-positive cocci in clusters (GPCC) by targeting* mecA* for determining methicillin resistance,* femA* specific for* S. aureus* (*femA*-SA),* femA *specific for* S. epidermidis* (*femA*-SE),* 16S rRNA* for universal bacteria, and* 16S rRNA* specific for staphylococci.

## 2. Materials and Methods

### 2.1. Blood Culture

This study evaluated 290 blood cultures containing GPCC obtained from March 2013 to December 2013 at Seoul National University Bundang Hospital. Two or more pairs of culture bottles for aerobes or anaerobes were incubated in BacT/Alert 3D (bioMérieux Inc., Durham, NC, USA) or BACTEC FX (BD Diagnostics, Sparks, MD, USA) blood culture systems for 5 days after inoculation for blood drawn from the patient. If bacterial growth was not detected within 5 days, then the blood culture result was considered negative. When bacterial growth was noted, blood from the positive bottles was Gram-stained, and samples containing GPCC (230 specimens in BacT/Alert 3D (bioMérieux Inc.) and 60 specimens in BACTEC FX (BD Diagnostics)) were inoculated onto blood agar plates and cultured overnight at 35°C in a 5% CO_2_ incubator. Isolates were identified by colony morphology, Gram-staining, catalase, and coagulase tests. Final identification according to phenotypic characteristics and antimicrobial susceptibility tests was performed using the MicroScan WalkAway system (Siemens Healthcare Diagnostics, Deerfield, IL, USA) with Pos Combo Panel Type 1A.

### 2.2. DNA Extraction

A 100 *μ*L aliquot of blood was drawn directly from the positive blood culture bottles, collected on filter paper, and dried for 15 min at room temperature. The blood spot was lysed with 2 mL of lysis buffer for 30 min at room temperature. Next, the paper was removed, and 2 *μ*L of lysozyme-Tris-EDTA buffer was added to 500 *μ*L of the eluate and incubated for 30 min at 37°C. The sample was incubated again for 10 min in 2 mL of lysis buffer, and the final eluate was used for nucleic acid extraction with the NucliSENS easyMAG platform (bioMérieux Inc.).

### 2.3. Multiplex Real-Time PCR Assay Targeting* mecA*,* femA-*SA,* femA-*SE, and Universal* 16S rRNA*

The double duplex real-time PCR TaqMan assay was performed with two tubes in one reaction. One tube corresponded to the targets* femA*-SA and* mecA* and the other to the targets* femA*-SE and universal* 16S rRNA*. The primers and probes for each target were designed as described by previously published studies [9, 10] (Table 1). Real-time PCR was conducted in a total volume of 20 *μ*L, including 2.0 *μ*L of 10x LightCycler FastStart DNA Master HybProbe (Roche Diagnostics, Mannheim, Germany), 2.4 *μ*L of 15 mM MgCl_2_ (Takara Bio, Shiga, Japan), 0.2 *μ*M of each primer, and 0.1 *μ*M of each probe with 3.0 *μ*L of DNA template. The amplification conditions used by the m2000rt instrument (Abbott Diagnostic, Chicago, IL, USA) were as follows: 95°C for 10 min followed by 35 cycles of 95°C for 15 sec and 60°C for 1 min in a single real-time PCR assay. Positive controls with MRSA, MSSA, and MRSE and a negative control with sterile distilled water (DW) were included throughout the procedures.

### 2.4. Additional Real-Time PCR Assay Targeting Staphylococcal* 16S rRNA*

A real-time PCR assay targeting staphylococcal* 16S rRNA* was performed using a LightCycler 2.0 system (Roche Diagnostics) for the detection of staphylococci. Primers targeting staphylococcal* 16S rRNA* were designed as described in a previously published study [11] (Table 1). Amplification reactions were performed in a 20 *μ*L volume containing 2 *μ*L of DNA template, 0.25 *μ*M of each primer, and 10 *μ*L of 2x SYBR Premix Ex Taq (Takara Bio). The conditions consisted of an initial denaturation at 95°C for 10 min followed by amplification program for 30 cycles of 10 sec at 95°C, 20 sec at 58°C, and 20 sec at 74°C with fluorescence acquisition at the end of each cycle. The amplification program was followed by a melting program consisting of heating to 95°C with a 0 sec hold and 15 sec at 60°C and a gradual increase to 99°C at a rate of 0.1°C/sec with fluorescence acquisition at each temperature transition. Positive controls with MRSA, MSSA, and MRSE and a negative control with sterile DW were included throughout the procedure. The existence of the target was confirmed by melting curve analysis. If the melting temperature (Tm) of the samples was within 0.5°C of the Tm of the positive control's product, they were regarded as positive.

All the above-mentioned real-time PCR assays were repeated with DNA extracted with subcultured colonies for comparison with the direct specimens.

### 2.5. Categorization of Real-Time PCR Results

Using a double duplex real-time PCR assay, the detection of* femA*-SA and universal* 16S rRNA* indicated the presence of MSSA, while the detection of* femA*-SA,* mecA*, and universal* 16S rRNA* indicated the presence of MRSA. If* femA*-SE and universal* 16S rRNA* were detected, the presence of MSSE was inferred, while the detection of* femA*-SE,* mecA,* and universal* 16S rRNA* indicated the presence of MRSE. The detection of* mecA* and universal* 16S rRNA* was interpreted as indicating the presence of MRCoNS or methicillin-resistant nonstaphylococci, while the detection of universal* 16S rRNA* alone was interpreted as indicating the presence of MSCoNS or methicillin-susceptible nonstaphylococci. The additional PCR test targeting staphylococcal* 16S rRNA* confirmed whether the isolate was staphylococci. The detection of universal* 16S rRNA*, but not staphylococcal* 16S rRNA*, was interpreted as indicating the presence of bacteria other than staphylococci (Table 2). The real-time PCR results were compared with MicroScan identification and susceptibility results as a reference.

## 3. Results 

### 3.1. Identification Results Obtained with the MicroScan WalkAway System (Siemens Healthcare Diagnostics)

Of the 290 positive blood cultures with GPCC, 262 cultures were identified to the species level by MicroScan as follows: 89* S. aureus*, 96* S. epidermidis*, 27* S. hominis*, 26* S. capitis*, 9* S. haemolyticus*, 5* S. capitis* subsp.* urealyticus*, 2* Staphylococcus saprophyticus*, 2* S. lugdunensis*, 2* S. hominis* subsp.* hominis*, 1* S. cohnii*, 1* S. auricularis*, 1* S. schleiferi*, and 1* S. schleiferi* subsp.* coagulans*. Fifteen isolates showed low-probability identification, with multiple possible staphylococcal species, and the remaining 13 isolates were nonstaphylococci, including 12* Micrococcus* species and 1* Enterococcus faecalis*.

### 3.2. Comparison of Results Obtained by Real-Time PCR with Direct Specimens and the Results of the MicroScan WalkAway System (Siemens Healthcare Diagnostics)

The real-time PCR identification of MRSA correlated with the MicroScan results for 46 out of 47 specimens. The discordant one was identified as MSSA by MicroScan. The real-time PCR identification of MSSA correlated with the MicroScan results for all 42 isolates.

Eighty-five out of 96 blood cultures identified as containing MRSE by real-time PCR were confirmed by MicroScan, while four isolates were identified as MRCoNS and two as MSSE. The MicroScan results for the remaining five isolates were low-probability identifications with multiple possible staphylococcal species that were all resistant to methicillin. The real-time PCR identification of MSSE correlated with the MicroScan results for seven out of eight isolates; the discordant isolate was identified as MSCoNS by MicroScan.

A total of 50 out of 58 blood cultures containing MRCoNS, as determined by real-time PCR, were confirmed to be MRCoNS with MicroScan, while one isolate was identified as MRSE and another as MSCoNS. The MicroScan results for the remaining six isolates were low-probability identifications with multiple possible staphylococcal species that were all resistant to methicillin. Eighteen out of 26 blood cultures containing MSCoNS by real-time PCR were confirmed by MicroScan, while three were identified as MRCoNS and one as MSSE. The MicroScan results for the remaining four isolates were low-probability identifications with multiple possible staphylococcal species that were all sensitive to methicillin. Thirteen isolates that were interpreted as nonstaphylococci by real-time PCR were confirmed as 12* Micrococcus* species and 1* Enterococcus faecalis *by MicroScan system.

For the 262 blood cultures identified to the species level by MicroScan, the results agreed very well with those obtained by real-time PCR for staphylococcal species categorization into MRSA, MSSA, MRSE, MSSE, MRCoNS, and MSCoNS (Cohen's unweighted kappa coefficient *κ* = 0.9313) (Table 3). The sensitivity and specificity of* mecA*,* femA*-SA,* femA*-SE, universal* 16S rRNA*, and staphylococcal* 16S rRNA* were evaluated with the 262 blood cultures according to the MicroScan results. The sensitivity and specificity were, respectively, 98.4% and 94.5% for the* mecA* gene, 100.0% and 100.0% for* femA*-SA, 97.9% and 97.0% for* femA*-SE, 100.0% and 100.0% for universal* 16S rRNA,* and 100.0% and 100.0% for staphylococcal* 16S rRNA *(Table 4).

### 3.3. Comparison of the Results Obtained by Real-Time PCR with Direct Specimens and Real-Time PCR with Subcultured Colonies

The real-time PCR results of direct specimens correlated with the PCR results of subcultured colonies for 282 of 290 samples. The results for 14 out of the 282 concordant samples were discordant with the MicroScan results. Of the seven samples that were interpreted as MRSE by both real-time PCR techniques, three were identified by MicroScan as MSCoNS, three as methicillin-resistant staphylococci (not identified to the species level), and one as MSSE. In two samples identified as MRCoNS by both real-time PCR techniques, the MicroScan identifications were MRSE and MSCoNS. Of the four samples identified as MSCoNS by both real-time PCR techniques, one was identified by MicroScan as MSSE and the other three were identified as MRCoNS. The remaining sample identified as MSSE by both real-time PCR techniques was identified as MSCoNS by MicroScan.

There were eight discordant results between real-time PCR with direct specimens and real-time PCR with subcultured colonies. In two samples that were identified as MSSA and MSSE by MicroScan, only real-time PCR with direct specimens detected* mecA*. In one sample, identified as MRCoNS by MicroScan, only real-time PCR with direct specimens detected* femA*-SE. In another sample, identified as MRSA by MicroScan, only real-time PCR with colonies failed to detect* femA*-SA. Finally, in the remaining four samples, which were identified as MRSE by MicroScan, only real-time PCR with colonies failed to detect* femA*-SE. The discordant results for the three methods are shown in Table 5.

## 4. Discussion

In this study, a multiplex real-time PCR assay that targets* mecA*,* femA*-SA,* femA*-SE, universal bacterial 16S rRNA, and staphylococci-specific 16S rRNA was developed and evaluated with clinical samples for the rapid identification of GPCC and determination of methicillin susceptibility. Overall, there was very good agreement between the real-time PCR with direct specimens and the MicroScan system for the 290 blood cultures, indicating reliable categorization as MRSA, MSSA, MRSE, MSSE, MRCoNS, MSCoNS, and nonstaphylococci.

Real-time PCR correctly identified all 46 positive blood cultures, which were confirmed as MRSA by MicroScan. Of the 43 blood cultures identified as MSSA by MicroScan, all but one were identified as MSSA by real-time PCR; the discordant culture was identified as MRSA by real-time PCR and as MSSA by additional PCR with subcultured colonies. This result could be due to nonspecific amplification of the* mecA* gene, but the presence of the* mecA* gene at a very low level in the positive blood bottle is also a potential explanation. There were no* S. aureus* isolates that were identified as* mecA* negative by real-time PCR but methicillin-resistant by MicroScan. It would be more troublesome if methicillin-resistant strains were not detected by PCR because these cases are likely to result in treatment failures.

In two out of 96 cultures identified as* S. epidermidis* by MicroScan, the* femA*-SE gene was not detected by real-time PCR with direct specimens or real-time PCR with subcultured colonies. As suggested by the manufacturers of MicroScan, we used a cut-off of 85% for identification at the species level as indicating a high probability and no additional tests required. However, previous studies have reported the misidentification of staphylococci by MicroScan, and misidentification by MicroScan is also a possibility in these cases [12, 13].

The* mecA* gene was detected by real-time PCR in all 86 blood cultures that were identified as MRSE by MicroScan. In two samples out of 10 blood cultures identified as MSSE by MicroScan,* mecA* was detected by real-time PCR. PCR testing using DNA from the subcultured colonies was positive for* mecA* in one sample but negative in the other sample. In the case of positive* mecA* identification by both PCR assays (direct specimen and subcultured colonies) but identification as methicillin-susceptible by MicroScan, there is a possibility of false oxacillin susceptibility results due to the heteroresistance phenomenon, which is a consequence of the complex regulation of the phenotypic expression of the* mecA* gene [14, 15].

Among the 57 isolates identified as MRCoNS by MicroScan, three were negative in* mecA* using real-time PCR directly from the blood culture bottles and additional PCR with subcultured colonies. This result could be attributed to false-negatives due to the possible limitations of PCR assays, such as the presence of PCR inhibitors. However, another possible explanation is a resistance mechanism other than* mecA*. Strains without the* mecA* gene can acquire methicillin resistance modification of normal PBP genes or overproduction of staphylococcal *β*-lactamase, resulting in methicillin resistance [16–18]. There were two discordant results out of 20 blood cultures identified as MSCoNS by MicroScan. In one sample,* mecA* was detected by both real-time PCR assays (direct specimen and subcultured colonies), while* femA*-SE genes were detected in the other sample by both real-time PCR assays.

For the fifteen staphylococci that were not identified to the species level by MicroScan, the* mecA* results were all in concordance with phenotypic methicillin resistance. And for the thirteen isolates identified as* Micrococci* and* Enterococci* by MicroScan, only universal* 16S rRNA* genes were detected by real-time PCR assays.

One limitation of this assay is that it cannot determine methicillin resistance of* S. aureus* when staphylococci other than* S. aureus* are also detected as mixed culture with positive* mecA*. Unlike conventional identification and susceptibility testing, multiplex real-time PCR assay is unable to indicate methicillin resistance to each of the staphylococci when it comes to mixed culture [19, 20]. It would be important to incorporate the multiplex real-time PCR assay alongside conventional identification and susceptibility testing.

In a clinical laboratory setting, the rapid and reliable identification of staphylococci and the determination of their methicillin susceptibility are important for effective antibiotic therapy and avoidance of the inappropriate use of glycopeptides [21]. Conventional identification and susceptibility testing of positive blood cultures based on phenotypic characteristics can take up to 48 h after the GPCC are recognized by Gram-staining. For prompt initiation with optimal antibiotic therapy, clinical laboratories are incorporating molecular diagnostic methods, such as real-time PCR assays, which provide rapid, sensitive, and specific detection of microbial pathogens within a few hours [22–24]. Recently, real-time PCR assays have been developed for the detection of staphylococci directly from positive blood culture bottles in clinical microbiology laboratories [25–33]. However, these assays are limited in that although they are able to rapidly identify staphylococci and determine methicillin susceptibility, they only allow the identification of staphylococci and cannot discriminate Gram-positive cocci other than staphylococci. We hypothesized that the addition of a PCR assay targeting staphylococcal* 16S rRNA* might mitigate this limitation and target both universal* 16S rRNA* and staphylococcal* 16S rRNA*. In this study, thirteen isolates that were recognized as GPCC by preliminary Gram-staining were confirmed as* Micrococci* and* Enterococci* by MicroScan system; without an assay targeting the staphylococci-specific 16S rRNA, they would have been interpreted as MSCoNS by real-time PCR.

## 5. Conclusions

The direct multiplex real-time PCR assay of positive blood cultures containing GPCC can provide essential information for prompt initiation of appropriate antibiotic treatment at the critical point of infection with a turnaround time of no more than 4 h, which includes 2 h for DNA extraction and 1.5 h for PCR. Further studies should evaluate the clinical outcome and benefit of using this rapid real-time PCR assay incorporated with conventional culture methods for glycopeptide antibiotic therapy in the case of positive blood cultures growing GPCC in clinical settings.

## Figures and Tables

**Table 1:** PCR primers and TaqMan probes for *mecA*, *femA* specific for *S. aureus *(*femA*-SA), *femA* specific for *S. epidermidis* (*femA*-SE), and universal *16S rRNA *and PCR primers for staphylococcal* 16S rRNA*.

Target genes	Sequence
*mecA*	5′-CATTGATCGCAACGTTCAATTT-3′
	5′-TGGTCTTTCTGCATTCCTGGA-3′
	5′-FAM-TGGAAGTTAGATTGGGATCATAGCGTCAT-TAMRA-3′

*femA*-SA	5′-TGCCTTTACAGATAGCATGCCA-3′
	5′-AGTAAGTAAGCAAGCTGCAATGACC-3′
	5′-JOE-TCATTTCACGCAAACTGTTGGCCACTATG-BHQ1-3′

*femA*-SE	5′-CAACTCGATGCAAATCAGCAA-3′
	5′-GAACCGCATAGCTCCCTGC-3′
	5′-JOE-TACTACGCTGGTGGAACTTCAAATCGTTATCG-BHQ1-3′

Universal *16S rRNA*	5′-TCCTACGGGAGGCAGCAGT-3′
	5′-GGACTACCAGGGTATCTAATCCTGTT-3′
	5′-FAM-CGTATTACCGCGGCTGCTGGCAC-TAMRA-3′

Staphylococcal *16S rRNA*	5′-GCAAGCGTTATCCGGATTT-3′
	5′-CTTAATGATGGCAACTAAGC-3′

**Table 2:** Interpretation of results by real-time PCR assays.

	*mecA*	*femA*-SA^a^	*femA*-SE^b^	Universal *16S rRNA*	Staphylococcal *16S rRNA*
MRSA	P^c^	P	N^d^	P	P
MSSA	N	P	N	P	P
MRSE	P	N	P	P	P
MSSE	N	N	P	P	P
MRCoNS	P	N	N	P	P
MSCoNS	N	N	N	P	P
Nonstaphylococci	P or N	N	N	P	N

^a^ *femA* specific for *S. aureus.*

^b^ *femA* specific for *S. epidermidis.*

^c^Positive.

^d^Negative.

**Table 3:** Comparison of results by real-time PCR with direct specimen and results by MicroScan WalkAway system (thirteen isolates of nonstaphylococci are excluded.).

MicroScan WalkAway system	Real-time PCR assays with direct specimen					
	MRSA^a^	MSSA^b^	MRSE^c^	MSSE^d^	MRCoNS^e^	MSCoNS^f^
MRSA	46	—	—	—	—	—
MSSA	1	42	—	—	—	—
MRSE	—	—	85	—	1	—
MSSE	—	—	2	7	—	1
MRCoNS	—	—	4	—	50	3
MSCoNS	—	—	—	1	1	18
Unidentified staphylococci^*∗*^	—	—	5	—	6	4

^*∗*^All the results of *mecA* were in concordance with phenotypic methicillin resistance.

^a^Methicillin-resistant *S. aureus*.

^b^Methicillin-susceptible *S. aureus.*

^c^Methicillin-resistant *S. epidermidis.*

^d^Methicillin-susceptible *S. epidermidis.*

^e^Methicillin-resistant non-*S. epidermidis* CoNS.

^f^Methicillin-susceptible non-*S. epidermidis* CoNS.

**Table 4:** Sensitivity and specificity of *mecA*, *femA* specific for *S. aureus *(*femA*-SA), *femA* specific for *S. epidermidis* (*femA*-SE), universal *16S rRNA*, and staphylococcal *16S rRNA*.

Target genes	Sensitivity (%)	Specificity (%)
*mecA*	98.4	94.5
*femA*-SA	100.0	100.0
*femA*-SE	97.9	97.0
Universal *16S rRNA*	100.0	100.0
Staphylococcal *16S rRNA*	100.0	100.0

**Table 5:** The discordant results between real-time PCR with direct specimen, real-time PCR with subcultured colonies, and MicroScan WalkAway system.

Real-time PCR with direct specimen	Real-time PCR with subcultured colonies	MicroScan WalkAway system	Number
MRSE^a^	MRSE	MRCoNS^b^	3
MRSE	MRSE	MSSE^c^	1
MRSE	MRSE	MR staphylococci	3
MSSE	MSSE	MSCoNS^d^	1
MRCoNS	MRCoNS	MRSE	1
MRCoNS	MRCoNS	MSCoNS	1
MSCoNS	MSCoNS	MSSE	1
MSCoNS	MSCoNS	MRCoNS	3
MRSA^e^	MSSA^f^	MSSA	1
MRSA	MRCoNS	MRSA	1
MRSE	MSSE	MSSE	1
MRSE	MRCoNS	MRCoNS	1
MRSE	MRCoNS	MRSE	4

^a^MRSE, methicillin-resistant *S. epidermidis.*

^b^MRCoNS, methicillin-resistant non-*S. Epidermidis* CoNS.

^c^MSSE, methicillin-susceptible *S. epidermidis.*

^d^MSCoNS, methicillin-susceptible non-*S. epidermidis* CoNS.

^e^MRSA, methicillin-resistant *S. aureus.*

^f^MSSA, methicillin-susceptible *S. aureus*.

## Additional Points

(i) We developed multiplex real-time PCR assay for rapid categorization of staphylococci. (ii) The targets were* mecA*,* femA*, universal* 16S rRNA,* and staphylococcal* 16S rRNA*. (iii) The results directly from positive blood culture bottles were compared with MicroScan. (iv) The assay rapidly detected methicillin-resistant staphylococci with good agreement.
